# Artificial Intelligence Aids in the Development of Multidisciplinary Age-Friendly Education

**DOI:** 10.1093/geroni/igaf122.2240

**Published:** 2025-12-31

**Authors:** Suzanne White, Robin McAtee, Laura Spradley

**Affiliations:** College of Nursing - University of Arkansas for Medical Sciences, Little Rock, Arkansas, United States; University of Arkansas for Medical Sciences, Little Rock, Arkansas, United States; University of Arkansas for Medical Sciences, Little Rock, Arkansas, United States

## Abstract

This work describes a structured approach to leveraging Artificial Intelligence (AI), specifically ChatGPT, a Large Language Model (LLM), to streamline and accelerate the development of educational content while maintaining accuracy and adherence to clinical best practices. Goals were developed to meet the learning objectives of the targeted learners and then AI was introduced. The process began by prompting ChatGPT to generate an e-learning module with specific instructional design elements based on goal-oriented outcomes while integrating age-friendly content into specialized geriatric modules for healthcare professionals. For example, the prompt for “Introducing the 4Ms of Age-Friendly Care” included: “Generate a structured 15-minute e-learning module introducing the 4Ms Framework to licensed and unlicensed healthcare professionals, 8th grade reading level, include an introductory paragraph, key sections with structured content, a conclusion paragraph, key takeaways, and a scenario-based application activity. Use content from the IHI website and the John A. Hartford Foundation only”. In seconds, ChatGPT generated the requested e-learning content. The output was reviewed and refined to ensure the desired competencies were appropriately addressed. Subject matter and educational experts conducted a final review to ensure accuracy, adherence to the desired instructional design parameters, and applicability to real-world healthcare settings. After the final review, ChatGPT was prompted to generate knowledge assessments to test key concepts. Key takeaways included: ChatGPT, when coupled with human expertise and oversight for educational content development, was a useful tool to streamline module creation, ensure alignment with instructional design principles, integrate age-friendly care elements, and generate knowledge assessments to evaluate learning.

